# Personality Unleashed: Surveying Correlation of Neuter Status and Social Behaviour in Mixed-Breed Male Dogs across Weight Classes

**DOI:** 10.3390/ani14162445

**Published:** 2024-08-22

**Authors:** Carina A. Kolkmeyer, Ana M. Zambrano Cardona, Udo Gansloßer

**Affiliations:** 1 Institut für Zoologie & Evolutionsforschung mit Phyletischem Museum, Ernst-Haeckel-Haus und Biologiedidaktik, Erbertstr. 1, 07743 Jena, Germany; udo@ganslosser.de; 2 Department of Biology, University of Vechta, Driverstrasee 22, 49377 Vechta, Germany; 3 Department of Biology, Chemistry, Pharmacy, Institute of Biology, Freie Universität Berlin, Altensteinstraße 6, 14195 Berlin, Germany; zambranoa96@zedat.fu-berlin.de

**Keywords:** neutering, castration, mixed-breed dogs, weight classes, questionnaire, behaviour, personality

## Abstract

**Simple Summary:**

Neutering is one of the most common procedures for dogs worldwide. While motivations for neutering vary, this procedure is often chosen with the hope of addressing undesirable behaviours in male dogs. In our study, 230 mixed-breed dogs (115 neutered and 115 intact) were examined. The dogs were categorised as “small”, “medium”, “large”, and “giant”. An online questionnaire was used, which asked about certain behavioural characteristics on the one hand and personality on the other. The results showed that neutered dogs appeared more stressed and aggressive than intact dogs. In addition, neutered small, medium, and large dogs were more stressed than giant dogs. Neutered dogs were also less social, trainable, emotionally calm and extroverted than intact dogs. This all suggests that neutering should be questioned and decided on an individual basis, as hormonal changes can have a considerable influence on dog behaviour.

**Abstract:**

This study investigates the behavioural differences between neutered and intact dogs. A total of 230 questionnaires of neutered (n = 115) and intact (n = 115) mixed-breed male dogs were collected. Small, medium, large, and giant dogs were analysed to investigate a possible influence of body size. The results showed neuters were significantly more stressed than intacts (multinomial logistic regression, *p* = 0.001). In terms of stress by influences (such as separation anxiety and anxiety at car rides), neutered small, medium, and large dogs were more stressed than giant dogs (multinomial logistic regression, *p* = 0.05). Neuters were also found to be more aggressive in general (multinomial logistic regression, *p* = 0.04) and more aggressive on the walk (multinomial logistic regression, *p* = 0.02). In addition, personality questionnaires revealed significant differences in all characteristics. Neuters were less emotionally calm (multiple ordinal regression, *p* = 0.03), less trainable and less sociable (each multiple ordinal regression, *p* < 0.001). They also scored lower on extraversion (multiple ordinal regression, *p* = 0.04). These findings highlight the importance of further research into the behavioural effects of neutering dogs, as well as the need for evidence-based guidelines for neutering practices.

## 1. Introduction

Neutering is a common technique across the world, although opinions on its advantages and disadvantages are still divided. Frequently cited reasons for neutering male dogs include mounting, hypersexuality, aggression, marking, or straying [1,2,3]. Recent studies have revealed that the advantages of neutering may not be as great as once thought and that it may even have detrimental consequences on a dog’s health and social behaviour [4,5,6]. According to some research, neutered dogs even exhibit more aggressive behaviour on a regular basis and experience greater levels of tension and anxiety [5,7].

Since neutering can lead to hormonal changes due to the loss of testosterone, these should be briefly included. On the one hand, neutering can lead to an imbalance of testosterone and cortisol, as cortisol competes with the sex steroid hormones for binding sites [8]. This imbalance can lead to increased anxiety, stress, or panic, which is confirmed by some studies [5,6,7,9,10,11,12,13,14,15]. On the other hand, the hormonal shift can lead to a decrease in oxytocin, as the oxytocin receptors are activated by the sex hormones [16]. After neutering, the sex hormones are absent, and it could be assumed that, consequently, less oxytocin can be bound.

Furthermore, this imbalance in oxytocin release can lead to more aggression [17,18]. The effect of neutering on the aggressive behaviour of dogs has been examined in several studies [19,20,21,22,23]. Remarkably, the elimination of testosterone has no effect on partner protection, which is regulated by vasopressin and oxytocin [17,24], or parental protection, which is regulated by prolactin [25]. While some studies assume an increase in aggressiveness [26,27,28,29], some research [30,31] suggested that aggressive behaviour decreases after neutering. For example, sexual competition between dogs, especially between males, might be reduced via neutering, but only to some extent by eliminating the sexual function, although this is not a guarantee [21,31].

Nevertheless, the topic of aggression is very complex, as it should always be noted that there are different types of aggression (like, e.g., defensive or status-related aggression) [26,27,28,29,32,33,34,35]. However, it is not just hormones that play a decisive role in the occurrence of certain forms of aggression. Body size has also been linked to behaviour in several past research [36,37], with smaller dogs being more prone to behave aggressively than large dogs.

Mikkola et al. [38] found that medium-sized and large dogs did not differ in their likelihood of displaying aggressive behaviour from small dogs. According to some previous research, larger and heavier dogs were shown to be less aggressive towards their keepers and strangers than smaller dogs [36], and Ley et al. [37] found that heavier dogs are more amiable than smaller dogs. Guy et al. [20], as well as Arhant et al. [39], also highlighted a potential link between lower body weight and fear and aggression. Similarly, Martínez et al. [40] found that smaller dogs displayed more aggressive behaviour compared to larger dogs. These findings support the association between small size and aggressive behaviour, whereas Bennett and Rohlf [41] found no correlation between a dog’s body size and aggressiveness or lack thereof. Khoshnegah et al. [42] observed that larger breeds exhibited more aggressive behaviour towards strangers. However, an important side note is that, for example, the study by McGreevy et al. [36] is based on breed standards and not on individual weight classes.

Nevertheless, large and small dogs can differ in their executive functions. Studies show that dog breeds with larger brains have better self-control and short-term memory than dogs with smaller brains. This indicates a positive correlation between the evolutionary increase in brain size and increasing executive function [43].

Prior research has also examined breed-specific variations in aggressive behaviour, and several studies have found noteworthy breed-specific variations [22,44,45,46]. Furthermore, some study findings show that the animal’s sex, breed, and age of neutering all significantly influence the consequences of the procedure [9,10,11,12,25]. In addition to age at the time of neutering and the dog’s previous experiences, individual characteristics and the dog’s breed can be decisive factors. However, Kolkmeyer et al. [10], among others, have shown that the influence of the dog breed is not as great as often assumed. In their questionnaire study, neutered dogs tended to display increased anxiety, stress, and aggression, irrespective of their breed.

In the follow-up study in 2024 [47], in which 68 huskies and 68 bulldogs (sensú Parker et al. [13] were examined, a similar trend emerged. Here, too, the neuters appeared more emotionally unstable and panicky. However, the effect was greater in the neutered bulldogs, so a breed-dependent effect can be assumed here. The aggression was reported in a higher proportion of neutered dogs, including more Huskies than Bulldogs, suggesting a potential breed-dependent influence in this aspect as well.

The aim of our study is to investigate differences in the social behaviour of neutered and intact male dogs. As mixed-breed dogs are a diverse and heterogeneous population reflecting a variety of genetic backgrounds, they were specifically selected as subjects. This makes it possible to analyse the effects of neutering on behaviour while minimising potential confounding factors associated with breed-specific traits.

Furthermore, mixed-breed dogs reflect a representative cohort, as they are found in many households. The American Veterinary Medical Association (AVMA; [48]) estimates that 53% and 44%, respectively, of all dogs residing in American households are mixed-breed dogs. About 31–33% of dogs in Germany and the UK are mixed breeds [49,50], while in Australia, half of all dogs living in human families are mixed breeds [51]. About one-third of dogs in scientific databases are mixed breeds [41,52].

Studying mixed-breed dogs, in particular, could also be interesting because they can exhibit behavioural differences from purebred dogs. Turcsán et al. [53] compared purebred with mixed-breed dogs and found that the latter are less calm and gregarious. They also showed significantly more problem behaviour overall than purebred dogs.

It is also conceivable that mixed-breed dogs, especially those from animal welfare, show many behavioural problems because there are many castrations for population control in these areas [54,55].

The results of Morrill et al. [56] also contribute to the decision to conduct a mixed-breed behavioural analysis. They stated that there are large differences in behaviour between individual dogs, even within breeds. This highlights the limitations of using breed as a predictor of the behaviour of the individual. Therefore, the wide size range within mixed-breed dog populations provides an additional opportunity to investigate the relationship between size and behaviour in more detail. This approach is particularly relevant considering recent research demonstrating relationships between morphology and behaviour in dogs [36,40,43,57].

Another important factor in the effects of neutering is the dog’s personality or character. Personality has become a focal point in behavioural ecology, defined by consistent individual differences in behaviour [58]. Personality encompasses the distinctive pattern of thoughts, feelings, and behaviours that differentiate one individual from another and remain consistent across various situations and over time [59]. In the context of dogs, it encompasses stable behavioural tendencies influenced by genetics, cognition, and environment, investigated through methodologies like behavioural assessments and questionnaires [37]. Personality in relation to neutering involves a complex interplay between behaviour changes and their lasting impact on a dog’s demeanour and well-being [60]. Despite neutering potentially leading to diverse behavioural alterations influenced by breed, age, and temperament, individual differences also shape dogs’ responses. These behavioural changes can contribute to shaping the dog’s personality over time, showcasing the bidirectional link between neutering and personality. In our analysis, the Budapest questionnaire developed by Turcsán et al. [61] was used. Neutered and intact mixed-breed dogs were examined to answer the following questions: (1) Are there any differences in stress-indicating or nervous behaviour between neutered and intact mixed-breed male dogs (in relation to body size)? (2) Do differences in aggressive behaviour occur between neutered and intact mixed-breed males (in relation to body size)? (3) Do neutered and intact mixed-breed male dogs show differences in the four personality traits (trainability, extraversion, sociability, and emotional stability (sensú Turcsán et al. [61]) (in relation to body size)?

## 2. Materials and Methods

An online structured questionnaire was used to conduct the analysis. The questionnaire was deployed using the “survio” online platform, and data were collected between December 2022 and March 2023. The purpose of the two-part questionnaire—the BUDAPEST questionnaire and the case studies—was to gather information from dog keepers. The exact items of the questionnaires can be found in the Supplementary Tables.

### 2.1. Case Studies

The case studies section of the questionnaire entailed questions about the dogs’ demographics, including age, gender, weight, and neuter status. The questionnaire, derived from the MAMMALIA AG counselling centre, has been utilised in various studies [5,6,10,47,62] and has proven to be a reliable assessment tool. The dog’s breed was indicated by the keepers, and no validation was asked for. The questions that followed focused on the dog’s origin, environment, and daily routine. Finally, there were inquiries concerning the issues with the cohabitation of dogs and any prior medical conditions. In the case studies, questions were mainly asked about stress-indicating behaviour, aggressive behaviour and nervous behaviour. When it came to stress, dog owners were asked whether their dog appeared stressed in certain situations. The questions were open-ended, and categories were formed regarding stress-inducing stimuli such as other people, other dogs, or noises, for example.

In the case of aggression, for example, specific questions were asked about whether the dog is aggressive on the leash, whether the dog is aggressive towards other dogs or whether there is aggressive behaviour towards humans. It was possible to tick whether the behaviour applied or not. Nervous behaviour was treated in a similar way. Here, too, the dog owners were able to tick which behaviour (nervous trembling, restlessness, never getting tired, etc.) applied to their dog (see Table 1).

The raw data in the Supplementary Materials show exactly which questions were included in the survey.

This section’s questions came in a range of formats, from selection lists to open-ended questions.

### 2.2. BUDAPEST Questionnaire

The BUDAPEST questionnaire, commonly referred to as DOGS Test [61] comes from a Hungarian research group [61]. It offers details on the following four dog personality traits: calmness (also known as emotional stability), extraversion, trainability, and sociability. In this study, “Extraversion” was selected instead of “Boldness” because “Boldness” has a different meaning in the behavioural ecology as the supertrait “bold” of the shy-bold-system sensú Taborsky et al. [63].

Each of the items has a three-point Likert scale for responses (0 being “Applies”, 1 being “Partially Applies”, and 2 being “Does Not Apply”). For some questions, the response scores had to be inverted. The total of the individual score values for each question was used to determine the final score for each personality trait.

The main contents of both questionnaires are summarised in Table 1.

### 2.3. Data Collection

The online questionnaire was distributed to friends and family and on a number of websites, such as Facebook groups and Instagram, in addition to dog schools and veterinarian clinics. The data collection process was designed to ensure anonymity, so no personal information about the dog keeper was used (“convenience sample”).

### 2.4. Data Preparation

Data preparation for statistical analysis was performed using Microsoft Excel (Version 2016). Based on average weight ranges from previous body weight-focused studies [64,65,66], the 230 male dogs surveyed had their body weights in kilograms divided into four established weight classes. Specifically, small dogs (S) were categorized as those weighing under 10 kg, medium-sized dogs (M) were classified as weighing between 10 and 20 kg, large dogs (L) were designated as those weighing between 20 and 30 kg and giant dogs (XL) were those over 30 kg. 

### 2.5. Statistical Analyses

The selected data were then prepared for statistical analysis, which was conducted using the software SPSS (IBM, Version 29). Non-parametric tests were chosen for the analysis since the data’s measurement level was ordinal.

The effects of neuter status and weight classes on aggressive as well as stress-indicating behaviour were examined by means of multinominal logistic regression. A multiple ordinal regression (with combined effects) was used to evaluate the impact of neuter status and weight group on the personality traits (emotional stability, extraversion, trainability and sociability). The last category (1 = yes, the characteristic applies) was always selected as the reference category.

To evaluate the strength of the correlation between the variables, the effect size was calculated. For combined effects (neuter status and weight class), Cramer’s V [67,68] was used. The ranges for interpreting the indices of this effect size according to Funder and Ozer [67] are the following:

r < 0.05—tiny

0.05 ≤ r < 0.1—very small

0.1 ≤ r < 0.2—small

0.2 ≤ r < 0.3—medium

0.3 ≤ r < 0.4—large

r ≥ 0.4—very large

We employed Odds Ratio by Chen et al. [69] for single effects with the subsequent bandwidth [70]:

Exp(B) < 1.68—very small

1.68 ≤ Exp(B) < 3.47—small

3.47 ≤ Exp(B) < 6.71—medium

Exp(B) ≥ 6.71—large

## 3. Results

A total of 230 dog keepers took part in the survey, including 115 neutered and 115 intact dogs. Of these, 21 neutered and 22 intact ones belonged to the small dogs category, 39 neutered and 38 intact to medium, 42 neutered and 37 intact to large category and 13 neutered and 18 intact to giant dogs. An overview of the samples is shown in Table 2.

**Figure 1:**
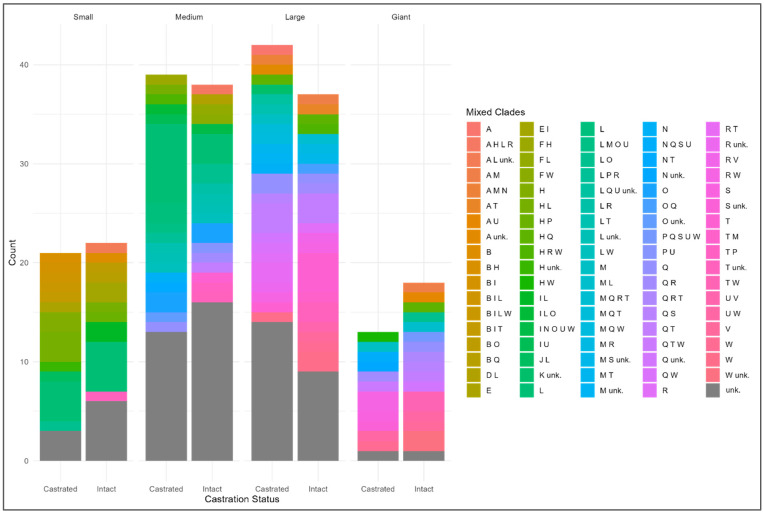
Distribution of clades combinations in the surveyed mixed-breed male dogs by neuter status and weight Class. Clades by Parker et al. [13]: A: Asian Spitz, B: Asian Toy, C: Nordic Spitz, D: Schnauzer, E: Small Spitz, F: Toy Spitz, G: Hungarian, H: Poodle, I: American Toy, J: American Terrier, K: Pinscher, L: Terrier, M: New World, N: Mediterranean, O: Scent Hound, P: Spaniel, Q: Retriever, R: Pointer Setter, S: Continental Herder, T: UK Rural, U: Drover, V: Alpine, W: European Mastiff.

The case studies allowed dog keepers to specify the supposed original breeds of their mixes where possible. The distribution of supposed dog breeds within the mixed-breed dogs is shown in Figure 1. To gather a better understanding of the possible breeds that constituted the mixed-breed male dogs in the analysis, the identified breeds were then systematically classified into their respective clades based on the methodology outlined by Parker et al. [13].

In the small weight class, the Terrier (L), Asian Toy (B), and Poodle (H) were prominent as constituent clades in many cases. Medium-sized dogs also frequently featured Terrier (L) clades, followed by Scent Hound (O), although unknown clades were highly present. Large dogs notably consisted of Retriever (Q) and UK Rural (T) clades, with unknown clades also being common. In the giant category, European Mastiff (W) and Retriever (Q) were the most notable clades.

**Figure 2:**
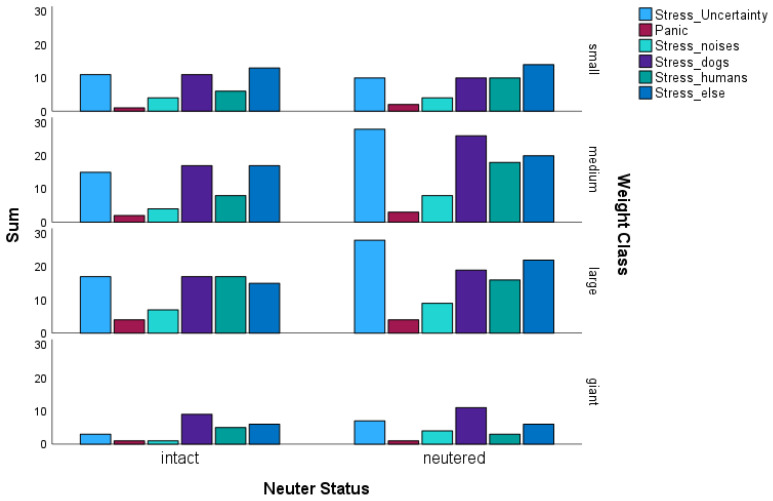
Bar chart of the stress-indicating behaviours of the N = 230 mixed-breed dogs (n = 115 neutered and 115 intact dogs). Stress/Uncertainty (n = 46 intact, n = 73 neutered dogs); Panic (n = 8 intact and 10 neutered dogs); Stress due to noises (n = 16 intact and 25 neutered dogs); Stress due to dogs (n = 54 intact and 66 neutered dogs); Stress due to humans (n = 36 intact and 47 neutered dogs); Stress due to anything else (n = 51 intact and 62 neutered dogs; else = separation anxiety, anxiety at the vet, car rides, new or unfamiliar environments). The vertical axis represents the sum of the number of dogs in which the characteristic occurred.

Among the 230 dogs surveyed, a total of 494 instances of stress-indicating behaviours were recorded for the six stress variables (see Figure 2). Out of these, 283 instances were exhibited by neutered males, while the remaining 211 instances were exhibited by intact males. The bar plot (Figure 2) provides insights into the frequency of stress-related behaviours among neutered and intact mixed-breed male dogs. In the diagram, it is noticeable that more neuters show stress and insecurity (n = 73) than intact dogs (n = 46). Statistical analyses revealed a significant *p*-value for both effects (neutering status and weight class; multinomial logistic regression; *p* = 0.001; Cramer’s V = 0.24). The main effect was primarily neuter status, which was significant (multinomial logistic regression; *p* < 0.001; OR = 0.38). Stress-related behaviour in relation to other dogs occurred just as frequently. Here, too, there were more neutered (n = 66) than intact (n = 54) dogs, but the difference was not significant. In addition, neutered dogs were more stressed in relation to humans (n = 47) than intact dogs (n = 36). There were also 25 neutered dogs that were stressed by noises, compared to 16 intact dogs. There were no significant differences between the neutered and intact mixed-breed dogs for either category or for panic.

However, there was a significant difference in stress with regard to anything else or other circumstances (multinomial logistic regression; *p* = 0.05; Cramer’s V = 0.16) for both variables. Here, the difference was found particularly along the weight classes (multinomial logistic regression; *p* = 0.05; OR = 1.32). The answers for stress because of other reasons were mainly separation anxiety, anxiety at the vet clinic, car rides, and new or unfamiliar environments.

In the category “hyperactivity/nervousness”, there was a trend for differences in hyperactive behaviour (multinomial logistic regression; *p* = 0.067). The trend was particularly noticeable for giant dogs, as they were less hyperactive than small, medium, and large dogs (multinomial logistic regression; *p* = 0.1; OR = 1.39).

Furthermore, 35 intact and 52 neutered dogs showed nervous behaviour, which was confirmed by a significant result (multinomial logistic regression; *p* = 0.02; OR = 0.53). There were no significant differences in trembling related to neutering status, but there were differences depending on the weight class (multinomial logistic regression; *p* = 0.06; OR = 1.35). The giant dogs in particular showed low values here compared to the other weight classes. There was a similar result for the variables “panting” (multinomial logistic regression; *p* = 0.07 for weight class; OR = 0.75) and “licking/scratching” (multinomial logistic regression; *p* = 0.05 for weight class; OR = 1.38).

**Figure 3:**
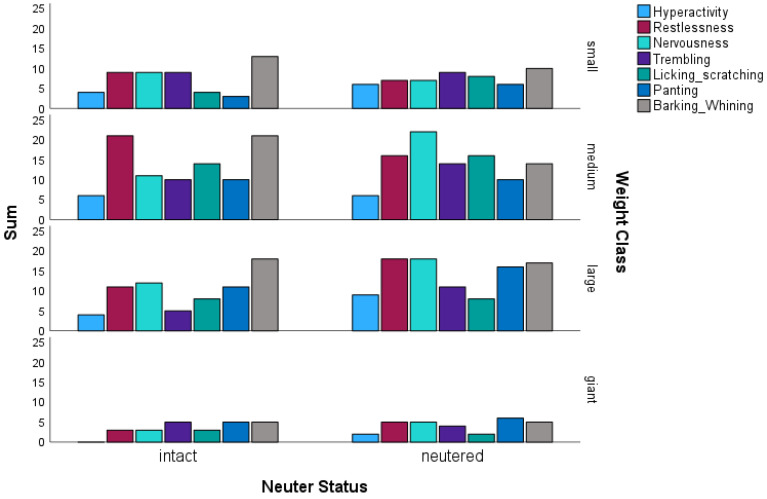
Comparison of hyperactive and nervous behaviour of neutered and intact mixed-breed dogs (N = 230) along the four weight classes. Hyperactivity (n = 14 intact and 23 neutered dogs); restlessness (n = 44 intact and 46 neutered dogs); Nervousness (n = 35 intact and 52 neutered dogs); Trembling (n = 29 intact and 38 neutered dogs); Panting (n = 29 intact and 38 neutered dogs); Licking/scratching (n = 29 intact and 34 neutered dogs); Barking/whining (n = 57 intact and 46 neutered dogs). The vertical axis represents the sum of the number of dogs in which the characteristic occurred.

In contrast to the above results, the characteristic “barking/whining” showed that there were more intact males than neutered males in which this occurred, but only with a slight trend (multinomial logistic regression; *p* = 0.08; Cramer’s V = 0.15). The results can be seen in Figure 3.

**Figure 4:**
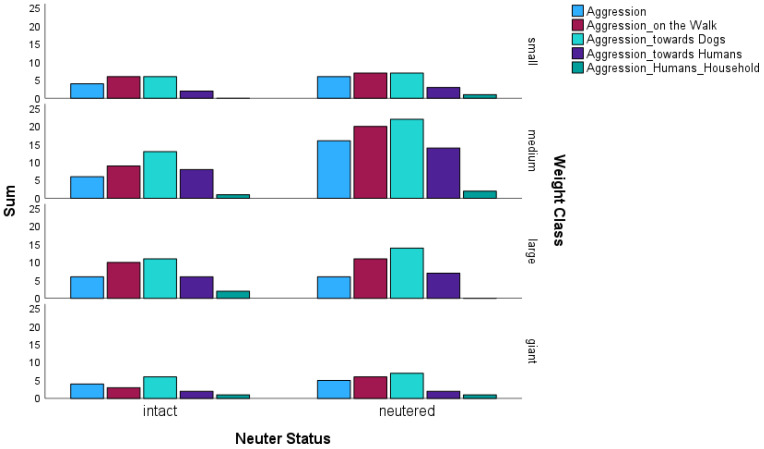
Comparison of the aggressive behavioural traits of the neutered and intact dogs (N = 230 dogs) in relation to their weight class. Aggression (n = 20 intact and 33 neutered dogs); Aggression on the walk (n = 28 intact and 44 neutered dogs); Aggression towards other dogs (n = 36 intact and 50 neutered dogs); Aggression towards humans (n = 18 intact and 26 neutered dogs); Aggression towards humans of the same household (n = 4 intact and 4 neutered dog). The vertical axis represents the sum of the number of dogs in which the characteristic occurred.

When comparing aggressive behaviour, it was noticeable that there were 157 cases for neutered dogs compared to 103 cases for intact dogs (see Figure 4). Neutered dogs differed significantly from intact dogs in terms of general aggression (multinomial logistic regression; *p* = 0.04; OR = 0.52). Aggression on walks was shown by 44 neutered and 28 intact dogs with a significant result (multinomial logistic regression; *p* = 0.024; OR = 0.52). There was a trend for aggression towards other dogs depending on neutering status (multinomial logistic regression; *p* = 0.057; OR = 0.59). Although there were no significant differences depending on the weight class, it was noticeable that small dogs also showed less general aggression (ca. 23% from all small dogs) than the other weight classes (28,9% at medium, 15% at large). It was striking that giant dogs showed the highest percentage of general aggression (29% of all giant dogs), despite the small sample size. Aggression towards other dogs was most prevalent in medium dogs (44.7%), followed by giant dogs with over 41%.

**Figure 5:**
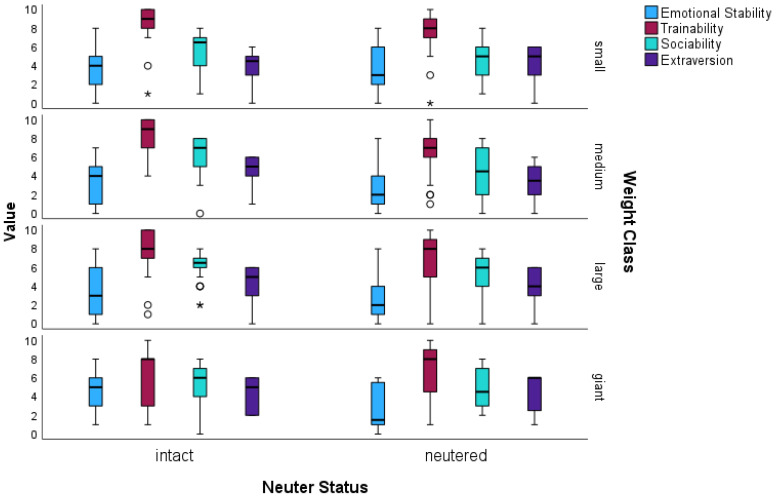
Boxplot of the 4 personality categories of the Budapest questionnaires (sensú Turcsán et al. [61]) of the 115 neutered and 115 intact mixed-breed dogs—depending on their weight class. Values that are more than 1.5 times the interquartile range away from the box are considered outliers and are shown as circles. Values outside these ranges are considered extreme values and are marked with an asterisk.

The results of the Budapest questionnaires (sensú Turcsán et al. [61]; Figure 5) showed that neutered dogs differed significantly from intact dogs in terms of emotional stability (multiple ordinal regression; *p* = 0.03; OR = 0.6), with lower values for neutered dogs (median = 2). The values of neutered dogs (median = 8) are also lower than those of intact dogs (median = 9) in terms of trainability, which can be confirmed statistically (multiple ordinal regression; *p* < 0.001; OR = 0.43). In addition, the dogs also differ from each other in terms of trainability depending on their weight (multiple ordinal regression; *p* = 0.024; OR = 0.7), with small dogs having higher values (median = 9) than the other weight categories (median for M, L and XL = 8 each). In terms of sociability, neutered dogs differed just as strongly from intact dogs, with a median of 5 compared to 7 for intact dogs. The ordinal regression analysis revealed a significant difference (multiple ordinal regression; *p* < 0.001; OR = 0.36). There was also a significant result for extraversion (multiple ordinal regression; *p* = 0.039; OR = 0.62) with more extroverted intact dogs (median = 5) than neutered dogs (median = 4). All statistically significant results from the case studies and Budapest questionnaire can be found in Table 3 and Table 4. In addition, all raw data from the responses to the questionnaires can be taken from the table in the Supplementary Materials.

## 4. Discussion

### 4.1. Stress-Indicating Behaviour

There is growing evidence correlating neutering in dogs with problematic behaviour such as stress, panic or aggression [5,6,7,10]. In our study, stress-indicating behaviour appears to be a problem for neutered dogs, as significantly more neutered than intact dogs showed stress or insecurity. One possible explanation can be found in the hormonal imbalance that can result from neutering, as already mentioned in the introduction [32,71]. However, it should be noted that cortisol primarily triggers the fight-or-flight response to a stressor [72]. It can, therefore, be assumed, as has also been confirmed in some studies, that increased cortisol levels can favour even more stress and intensify the response to it [73,74].

Although some researchers like Sandri et al. [75] found lower cortisol levels in neutered male and female dogs than in intact dogs, the effect of the decreased hormone levels is not yet sufficiently clear.

In our study, we also noticed that neuters are more stressed for various other reasons. Frequently mentioned were separation anxiety, anxiety at the vet clinic, car rides, and new or unfamiliar environments. Dinwoodie et al. [76] came to similar conclusions. Neuters of both sexes had significantly more fear or anxiety and also showed more escaping or running away. Increased separation anxiety in neuters is confirmed by some studies [77,78,79,80]. Neutered dogs are sometimes three times more likely to suffer from separation anxiety than intact dogs. Interestingly, mixed-breed dogs, in particular, appear to have a high risk of separation anxiety compared to purebred dogs [80,81].

Additionally, there were significant differences in sensitivity to stressed behaviour across the weight classes. It was evident that it mainly affected neutered medium and large dogs, which also tended to be more stressed as a percentage. More than every second dog in the medium and large class seemed to suffer from this behaviour.

A look at the main breeds that were present within these two weight classes shows that they mainly included representatives of the clades “Terrier”, “Retriever”, “UK Rural” (such as Australian Shepherd or Border Collie) and “Pointer Setter” (e.g., German wirehaired Pointer) (sensú Parker et al. [13]). According to Turcsán et al. [61], Australian Shepherds, Border Collies, Jack Russell Terriers, Wirehaired Pointers and Golden Retrievers, for example, are categorised as low calm.

Pastore et al. [82] studied the stress behaviour of agility dogs (especially Border Collies or mixes thereof) and came to the conclusion that these energetic dog breeds need to learn a large amount of impulse control in order to counteract the numerous stressors around them. For example, a Border Collie must be able to cross a tunnel without seeing the exit. Many of these dogs only master this as they get older, so most (younger) dogs are often more stressed as a result [82].

An extremely important aspect of mixed-breed dogs is that they can often have different socialisation than purebred dogs and can, therefore, already be emotionally pre-stressed, as some mixed-breed dogs come from local or foreign animal shelters [83]. The proportion of mixed-breed dogs in animal shelters is often very high. Salman et al. [84] found that 65% of dogs in shelters were mixed-breed dogs. Twenty years later, Luescher & Medlock [83] report much higher numbers and assume that 80% of the dogs in animal shelters are mixed breeds. Added to this is the high number of dogs from foreign animal welfare organisations, which are imported dogs [85].

One reason for this could be that puppies from unwanted litters often end up in shelters or become street dogs. Such unwanted offspring rarely occur in dogs of the same breed, so the puppies are more likely to be mixed breeds. If these animals end up in an animal shelter, they are exposed to a completely different environment than dogs that grow up in a private household [86,87]. There are some studies that clearly indicate a link between stress factors from the shelter (such as social and spatial confinement) and behavioural problems [88,89,90]. At this point, particular attention should be paid to the extent to which gonadectomy could be associated with possible stress, especially in the case of certain (mixed) dog breeds.

### 4.2. Nervous/Hyperactive Behaviour

When comparing hyperactive behaviour, there was a trend that giant dogs were less hyperactive than the other weight classes. Although it should be noted that there were significantly fewer dogs in the XL group than in other weight classes, this trend is not negligible. In percentage terms, 93.5% of giant dogs were classified as non-hyperactive and only 6.5% as hyperactive. In contrast, approximately 16% of large dogs and about 15% of medium dogs were categorised as hyperactive. In the case of small dogs, it was over 23%, so more than one in five dogs.

This can possibly also be explained by breed-related influences. When looking at the breeds that are predominantly found in small dogs, it is noticeable that some terriers (such as Jack Russell Terriers) are among them. These are more frequently categorised as hyperactive due to their breed [91]. In Sulkama et al. [91], it was mainly the Cairn Terriers or Jack Russell Terriers that appeared to be particularly hyperactive. In addition to Terriers, there were also many Chihuahua and Maltese mixes among small dogs. However, Chihuahuas are described as rather calm and less active [91], but a closer look at the Chihuahua mixes from our study reveals that they are most frequently mixed with clade L (“Terrier”) or clade U (“Drover” like, e.g., Giant Schnauzer, Doberman Pinscher) (sensú Parker et al. [13]).

In addition to breed-related effects, there are indications that the energy levels of dogs could also depend on their body size. Coren [92] found size-dependent differences in the activity of dogs. It was noticed that the energy level of dogs decreased with increasing body size. Giant dogs, in particular, had a very low energy level, which also corresponds to our results.

When comparing neutering status, there was only a trend for differences in barking/whining, and there were more intact dogs than neutered dogs who were barking. In Pongrácz et al. [93], neutered dogs whined/barked earlier, but overall, intact dogs also whined more than neutered dogs.

Barking is based on different motivations, whereby a distinction is made between different tonal sounds. A sonorous (tonal) bark often occurs in a playful context. Noisy barking, on the other hand, is categorised in a distance-extending context. Barking can also occur in combination with other sounds, such as growling and barking, howling and barking, or in conjunction with squeaking and crying. However, barking sounds vary depending on the individual and the breed [94]. Male and female dogs use barking for territorial defence, among other things [95].

### 4.3. Aggressive Behaviour

While examining the survey data regarding aggression, a notable difference was noted for general aggression between neutered and intact dogs, with more neutered dogs showing aggression than intact dogs. The higher instances of aggressive behaviour in neutered male dogs coincide with the results from Kaufmann et al. [5] and Farhoody and Zink [7]. Here, the researchers also found that neutered males are more likely to present aggressive behaviour than intact males.

An increased aggression in neutered male dogs could be linked to a hormonal imbalance of cortisol and testosterone [8,32,71] or altered oxytocin levels due to the loss of sex hormones [16,33,60,96]. Fluctuations in the serotonin balance are also possible [96,97,98,99,100].

Numerous experts in animal behaviour point out that a significant percentage of dogs displaying aggressive behaviour also exhibit symptoms of stress and anxiety [101,102]. Fear-driven aggression, in particular, is controlled by cortisol [98]. In addition, more neuters than intact dogs showed aggression on walks and aggression towards other dogs. This also corresponds to the studies mentioned above.

In addition to the studies that have found a negative effect of neutering, there are also studies that have observed either no effect [15,19,23] or even positive effects [103,104].

When comparing weight classes, however, it is noticeable that it was mainly neutered medium and giant dogs that were generally more aggressive (29% of all medium and giant dogs were aggressive, 23% of the small dogs and only 15% of the large dogs). Since the exact breed- and size-dependent effects of aggression have not yet been clarified [40,64], it is only possible to make assumptions.

There is also evidence, however, that there are dogs with a disproportion between brain size and body size, which has been encouraged by domestication. This extended brain development with expansion in distributed subcortical regions may be associated with behavioural problems (e.g., fear, aggression) [105].

When looking at the breeds, it is noticeable that there is great variability within giant dogs and that many clades of Parker et al. [13] are represented. These mainly include German Shepherd, Doberman, Golden Retriever, Australian Shepherd, Boxer and Large Poodle mixes. However, there are also some ancient dog breeds such as Chow Chow, Shiba Inu, and Huskies. It could possibly be argued here that especially ancient dog breeds are often described as more aggressive than more modern dog breeds [106].

The study by Goodwin et al. [107] found that paedomorphism in dogs, in particular, plays a role in the extent to which wolf-like behaviour is shown. More modern dog breeds, such as French Bulldogs or Cocker Spaniels, tend to show less wolf-like behaviour. On the other hand, breeds such as the Siberian Husky or the German Shepherd, which are closer to the original type [13], show more wolf-like behaviour. These behavioural differences are the result of heterochronic changes [108]. According to Goodwin et al. [107], dog breeds that are similar to juvenile wolves show less wolf-agonistic behaviour later in life.

In total, the majority of both neutered and intact dogs being more aggressive belong to the small and medium categories, which mainly include dog breeds from the clades such as “Terriers”, “Poodles”, “Toy Spitz” (e.g., Papillon), or “American Toy” (e.g., Chihuahua). Here, some studies describe smaller dog breeds as being more aggressive [36,37,39]. These results also coincide with the findings of Guy et al. [20], who emphasised that lower body weight was associated with a history of aggression, suggesting a possible link between fear and aggression in small dogs. Similarly, Martínez et al. [40] found that smaller dogs showed more aggressive behaviour compared to larger dogs. In contrast, Baranyiová et al. [64] found no correlation between the aggressiveness of dogs and their body size.

According to McGreevy et al. [36], there are some reasons why problematic behaviours are more common in smaller dogs: (1) Keepers of small dogs may be more relaxed about these behaviours; (2) The environment for small dogs may trigger such behaviours; (3) Selection for small size may lead to neurological changes that make small dogs more reactive; (4) Artificial selection for neotenous characteristics in small dogs. Overall, mixed-breed dogs appear to have a higher risk of fear-related aggression in particular [109].

Nevertheless, many factors are relevant to aggressiveness, like multi- or single-dog households [22], attachment to their keeper [22,110], or the time of weaning [111].

### 4.4. BUDAPEST Questionnaire

Regarding the BUDAPEST questionnaires within our study, we found that neuters are less emotionally calm than intact dogs. This is consistent with previous studies. Kaufmann et al. [5], Kolkmeyer et al. [10] and Lorenz et al. [6] also observed lower emotional stability in neutered dogs. One possible explanation could be the hormonal changes after neutering. Oxytocin, in particular, has an effect on a dog’s emotional mood. As this can also fluctuate after neutering [112], a negative effect on emotional reaction is possible.

For trainability, intact dogs had higher scores than neuters, which is in line with other studies [10,47,52]. Trainability refers to the openness of a dog. Low scores in this trait indicate a less playful and inventive character. Dogs with low scores are considered less open and inquisitive [52,61]. The assumption could be that neuters show altered behaviour due to hormonal changes and might lack motivation or calmness for playful activities. On the other hand, neutering can also lead to cognitive impairments such as disorientation [113,114,115,116]. Nevertheless, many factors influence trainability. In Kubinyi et al. [52], for example, dogs that took part in professional training courses were considered to be highly trainable. Daily interactions with the dog also increase trainability [59,117].

In addition to neutering status, dogs also differed in their trainability depending on their weight class. Small dogs were significantly more trainable than larger mixed-breed dogs. As already mentioned, small dogs included mainly Terrier and Poodle mixes, which, according to Turcsán et al. [61] and Serpell and Hsu [117], are also classified as very trainable.

When comparing body size regardless of breed, the life history development of smaller dogs could also have an impact on trainability. On average, they have a longer life expectancy [118], although they have a faster growth rate [66,119,120]. Larger dogs are, therefore, in a growth period for longer, and it is also assumed that once they start ageing, they age faster than smaller dogs [121].

However, while comparing mixed-breed dogs with purebred dogs, Turcsán et al. [53] showed that mixed-breed dogs are more trainable but less calm than purebred dogs. They also show problematic behaviour more frequently. Interestingly, more mixed-breed dogs were neutered than purebred dogs, which could possibly be an additional effect.

### 4.5. Limitations

This study has to be seen in light of some limitations. Although the questionnaires were validated, the results are based on the dog keepers’ assessments, an aspect that must always be taken into account.

The unequal sample distribution is also an important factor. There were fewer giant dogs than dogs from other weight classes. In addition, the cladogram of breeds must take into account that the occurrence of certain breeds within the mixed-breed dogs is only based on statements by the dog keepers. No pedigrees, breeding certificates, photos, or similar were requested. There is also the possibility that some of the dogs are not direct mixed breeds or hybrids but so-called “mutts” as described by Morrill et al. [56].

It would be interesting for future studies to enquire more specifically in the case studies about, for example, the dogs to whom aggression is directed in order to obtain even more detailed information about the action/reaction of dogs towards other dog sizes or breeds. In this study, the age of the dogs was not included in the statistical results because the sample size was too small to obtain meaningful results. It would, therefore, be exciting for further studies to include the age of the dogs in the statistical analyses in order to cover age-dependent factors.

## 5. Conclusions

In conclusion, our study shows a clear tendency for neutered mixed-breed dogs to differ from intact mixed-breed dogs. Particularly because mixed-breed dogs can often be prone to problematic behavioural problems, special care should be taken when neutering them. In addition to neutering-related differences, weight-related differences were also found, so neutering should also be carefully considered in relation to the dog’s size.

## Figures and Tables

**Table 1:** Budapest questionnaire and case studies.

Stress	Nervousness	Aggression	Budapest Questionnaire
uncertainty	licking/scratching	aggression in general	emotional stability
noises	seems absent	on the walk	trainability
dogs	never getting tired	towards dogs	extraversion
humans	restlessness	humans	sociability with dogs
panting	unreasonably nervous	humans of the same household	
stereotypic behaviour			
other			

**Table 2:** The sample distribution of the participating dogs along the four weight classes.

Weight Class	Neutered	Intact	Total
S (<10 kg)	21	22	43
M (10–20 kg)	39	38	77
L (>20–30 kg)	42	37	79
XL (>30 kg)	13	18	31
Total	115	115	230

**Table 3:** Results of multinomial logistic regression of the case studies with all effect sizes (Cramer’s V and Odd’s ratio (OR, Exp(B)). The last category (1 = yes, the characteristic applies) was always selected as the reference category.

	*p*-Value (Both Effects)	*p*-Value (Neuter Status)	*p*-Value (Weight Class)	Cramer’s V (Both)	Exp(B) (Neuter Status)	Exp(B) (Weight)
Stress/Insecurity	0.001	<0.001	n.s.	0.24	0.38	1.14
Stress (else)	0.05	n.s.	0.05	0.16	0.68	1.32
Hyperaktivity	0.07	n.s.	0.1	0.15	0.56	1.39
Nervousness	0.04	0.02	n.s.	0.17	0.53	1.16
Trembling	0.07	n.s.	0.06	0.15	0.69	1.35
Panting	0.08	n.s.	0.07	0.15	0.67	0.75
Licking/scratching	n.s.	n.s.	0.05	0.14	0.81	1.38
Barking/whining	0.08	n.s.	n.s.	0.15	1.49	1.28
Aggression	n.s.	0.04	n.s.	0.14	0.52	1.07
Aggression on the walk	0.06	0.02	n.s.	0.16	0.52	1.09
Aggression towards other dogs	n.s.	0.06	n.s.	0.13	0.59	0.97

**Table 4:** Results of multiple ordinal regression of BUDAPEST questionnaire (sensú Turscan et al. [61]) with all effect sizes (Cramer’s V and Odd’s ratio (OR, Exp(B)). The last category (1 = yes, the characteristic applies) was always selected as the reference category (n.s. = not significant).

	*p*-Value (Both Effects)	*p*-Value (Neuter Status)	*p*-Value (Weight Class)	Cramer’s V (Both)	Exp(B) (Neuter Status)	Exp(B) (Weight Class)
Emotional Stability		0.027	n.s.	0.15	0.6	0.97
Trainability	<0.001	<0.001	0.024	0.28	0.43	0.7
Sociability	<0.001	<0.001	n.s.	0.28	0.36	1.05
Extraversion	0.083	0.039	n.s.	0.14	0.62	1.11

## Data Availability

The raw data presented in this study are also available on request from the corresponding author.

## Supplementary Materials

The following supporting information can be downloaded at https://www.mdpi.com/article/10.3390/ani14162445/s1, Table S1: Case Srudies 1; Table S2: Case Studies 2; Table S3: Budapest Questionnaire
