# Supplementary material for: Personality Unleashed: Surveying Correlation of Neuter Status and Social Behaviour in Mixed-Breed Male Dogs across Weight Classes
Source: Animals (Basel). 2024 Aug 22;14(16):2445. doi: 10.3390/ani14162445 (PMC11350830; doi:10.3390/ani14162445)
Supplement: Supplementary file 1 [file animals-14-02445-s001.zip › animals-3132652-supplementary.pdf]

Case studies

Neuter Status	weight (kg)	weight class	Epilepsy	Thyroid	Stress_Uncertainty	Panic	Hyper-activity	Aggression	Stress_noises	Stress_dogs	Stress_humans	Stress_else	Aggression_Walk	Restless-ness	Never getting tired	nervous	Seems absent
neutered	3,0	S	0	0	0	0	0	0	0	0	1	1	0	0	0	0	1
neutered	3,0	S	0	0	0	0	0	0	0	0	0	1	0	0	0	0	0
intact	3,1	S	0	0	0	0	0	0	0	0	0	1	0	0	0	0	0
neutered	3,9	S	0	0	1	0	0	0	0	1	1	1	1	1	0	1	0
neutered	4,0	S	0	0	1	1	0	0	1	0	1	1	0	0	0	0	0
neutered	4,0	S	0	0	0	0	0	0	0	0	1	1	0	0	0	0	0
intact	4,3	S	0	0	1	1	1	0	0	1	0	1	0	1	1	1	0
intact	5,0	S	0	0	0	0	0	0	0	0	0	1	0	0	0	0	0
neutered	5,0	S	0	0	1	0	0	1	0	1	1	1	1	0	0	0	0
intact	5,0	S	0	0	1	0	0	0	0	0	1	0	0	0	0	0	0
intact	5,0	S	0	0	0	0	0	0	1	0	0	1	0	0	0	0	0
neutered	6,0	S	0	1	1	0	0	1	0	1	0	0	1	0	0	0	0
neutered	6,0	S	0	0	1	0	1	1	0	1	1	0	1	1	0	1	0
intact	6,0	S	0	0	1	0	1	0	0	0	1	1	0	0	1	1	0
neutered	6,0	S	0	0	0	0	1	0	0	0	1	1	1	1	0	1	0
neutered	6,0	S	0	0	1	1	1	1	0	1	0	0	1	1	1	1	0
intact	6,0	S	0	0	1	0	0	0	0	0	0	1	0	1	0	0	0
intact	7,0	S	0	0	0	0	0	0	0	0	0	0	0	0	0	0	0
intact	7,0	S	0	0	0	0	0	0	0	0	1	0	0	0	0	0	0
neutered	7,0	S	0	0	0	0	0	0	0	0	0	1	0	0	0	0	1
intact	7,0	S	0	0	1	0	1	0	1	1	1	1	0	1	1	1	0
intact	7,0	S	0	0	0	0	0	0	0	0	0	1	0	1	0	0	1
neutered	7,0	S	0	0	0	0	1	0	0	0	0	1	0	0	0	0	0
intact	7,2	S	0	0	0	0	0	0	0	0	0	0	0	0	0	1	0
neutered	7,3	S	0	0	0	0	0	0	1	0	0	1	0	0	0	0	0
intact	7,5	S	0	0	1	0	0	0	0	1	1	1	1	0	0	1	0
intact	7,6	S	0	0	1	0	0	0	0	1	0	0	0	0	0	0	0
neutered	7,8	S	0	0	0	0	0	0	0	0	0	0	0	0	1	0	0
intact	8,0	S	0	0	1	0	0	0	0	0	0	1	1	1	1	1	0
neutered	8,0	S	0	0	0	0	0	0	0	0	0	1	0	0	1	0	0
neutered	8,0	S	0	0	1	0	1	1	1	1	1	1	0	1	1	1	1
neutered	8,1	S	0	0	0	0	1	1	0	1	1	0	1	1	0	1	0
intact	8,2	S	0	0	0	0	0	1	1	1	0	1	1	1	0	0	0
intact	8,5	S	0	0	1	0	1	0	0	1	1	1	0	1	0	0	1
intact	8,9	S	0	0	1	0	0	1	1	1	0	0	1	0	0	1	0
neutered	9,0	S	0	0	0	0	0	0	0	1	0	0	0	0	0	1	0
intact	9,0	S	0	0	1	0	0	1	0	1	0	0	1	1	0	0	1
intact	9,0	S	0	0	0	0	0	0	0	1	0	1	0	0	0	0	0
neutered	9,0	S	0	0	1	0	0	0	1	1	0	1	0	0	0	0	0
intact	9,5	S	0	0	0	0	0	1	0	1	0	0	0	0	0	1	0
neutered	9,5	S	0	0	1	0	0	0	0	0	1	0	0	1	0	0	0
intact	9,6	S	0	0	0	0	0	0	0	1	0	0	1	1	0	1	0

neutered	9,8	S	0	0	1	0	0	0	0	1	0	1	0	0	0	**0**	0
neutered	10,0	M	0	0	0	0	0	0	1	0	0	1	0	0	0	0	0
intact	10,0	M	0	0	0	0	0	0	0	0	0	1	0	1	0	**0**	0
intact	10,0	M	0	0	0	0	0	0	0	0	0	1	0	0	0	**0**	0
intact	10,0	M	0	0	0	0	0	0	0	0	0	0	0	0	0	**0**	0
neutered	10,0	M	0	0	0	0	0	1	0	1	0	0	1	1	1	**0**	0
neutered	10,0	M	0	0	0	0	0	1	0	1	0	0	1	1	0	**1**	1
neutered	10,0	M	0	0	1	0	0	1	1	1	1	1	1	1	1	**1**	1
neutered	10,0	M	1	0	0	0	1	1	0	0	0	1	1	1	0	**1**	1
intact	11,0	M	0	0	0	0	0	0	1	0	1	0	0	0	0	0	0
intact	11,0	M	0	0	0	0	0	0	0	0	0	0	0	0	0	**0**	1
neutered	11,0	M	0	0	1	0	0	1	1	0	1	0	1	0	1	**1**	0
intact	11,0	M	0	0	1	0	0	1	0	1	0	0	1	1	0	**1**	0
neutered	11,0	M	0	0	0	0	0	0	0	0	0	1	0	0	0	**0**	1
intact	11,0	M	0	0	1	0	1	0	0	1	0	1	0	1	1	**1**	0
intact	11,0	M	0	0	0	0	1	0	0	0	0	1	0	1	1	**0**	0
neutered	11,5	M	0	0	1	0	1	0	0	1	0	0	0	1	1	1	0
intact	12,0	M	0	0	0	0	1	0	0	0	0	0	0	1	1	**0**	0
neutered	12,0	M	0	0	1	0	0	1	1	1	1	1	1	0	0	**1**	1
neutered	12,0	M	0	0	1	0	0	1	1	0	0	0	1	0	0	**0**	0
intact	12,0	M	0	0	0	0	0	0	1	0	0	0	0	0	0	**0**	0
neutered	12,0	M	0	0	0	0	0	0	0	0	1	0	0	0	0	**0**	1
intact	12,0	M	0	0	0	0	0	0	0	1	0	0	0	1	1	**0**	0
neutered	12,0	M	0	0	1	0	0	1	0	1	0	0	1	0	1	**1**	0
neutered	12,0	M	0	0	1	0	0	0	0	1	0	0	1	0	0	**0**	0
intact	12,0	M	0	0	1	0	0	0	0	0	0	1	0	0	0	**1**	0
neutered	13,0	M	1	0	1	0	0	1	0	1	0	0	1	1	0	1	0
neutered	13,0	M	0	0	1	0	0	0	0	1	1	0	0	1	0	**0**	0
intact	13,0	M	0	0	1	0	0	1	0	1	1	1	1	1	0	**1**	0
intact	13,0	M	0	0	0	0	0	0	0	1	0	0	0	0	0	**0**	0
intact	13,0	M	0	0	0	0	0	1	0	1	0	0	1	0	1	**0**	0
intact	13,0	M	0	0	1	1	0	0	0	0	0	1	0	1	0	**0**	0
intact	13,0	M	0	0	1	0	0	0	0	1	0	1	0	0	0	**0**	0
intact	13,5	M	0	0	0	0	0	0	0	0	0	0	0	0	0	**0**	0
intact	13,5	M	0	0	0	0	0	0	0	0	0	0	0	1	0	**0**	0
neutered	14,0	M	0	0	1	1	0	0	0	0	1	1	0	1	0	0	0
neutered	14,0	M	0	0	1	0	1	1	0	1	1	1	1	1	0	1	0
neutered	14,0	M	0	0	1	0	0	0	1	1	0	1	0	1	0	**1**	0
neutered	14,0	M	0	0	1	0	0	0	0	1	1	1	0	0	0	**0**	1
intact	14,0	M	0	0	0	0	0	0	0	0	0	0	0	0	0	**0**	0
neutered	14,5	M	0	0	1	0	1	0	0	0	0	1	0	1	1	**0**	0
intact	14,5	M	0	0	1	0	1	0	0	1	0	1	0	1	1	**1**	0
intact	15,0	M	0	0	0	0	0	0	0	0	0	0	0	0	0	**0**	0
neutered	15,0	M	0	0	0	0	0	0	0	1	0	1	1	0	0	**0**	0
neutered	15,0	M	0	0	0	0	0	0	0	0	0	0	0	0	0	**1**	0
intact	15,1	M	0	0	1	0	0	0	0	1	0	1	0	1	1	**1**	0
neutered	16,0	M	0	0	0	0	0	0	1	1	0	1	1	0	0	1	0

neutered	16,0	M	0	0	1	0	0	0	0	1	1	0	0	0	0	0	0
intact	16,0	M	0	0	1	0	0	1	0	1	0	1	1	1	0	1	0
neutered	16,0	M	0	0	1	0	0	0	0	1	0	0	0	0	0	**0**	1
neutered	16,0	M	0	0	1	0	0	1	0	1	0	0	1	0	0	**0**	0
intact	17,0	M	0	0	0	0	0	0	0	1	1	0	0	1	1	**0**	0
intact	17,0	M	0	0	1	0	0	1	0	0	1	1	1	1	0	**1**	0
intact	17,0	M	0	0	1	0	0	0	0	1	0	0	0	1	1	**0**	0
neutered	18,0	M	0	0	1	0	0	1	0	0	1	1	0	0	0	1	0
intact	18,0	M	0	0	0	0	0	0	0	0	0	1	0	0	0	0	0
neutered	18,0	M	0	0	0	0	0	0	0	0	1	0	0	0	0	0	0
intact	18,0	M	0	0	1	0	0	0	0	1	1	0	1	1	1	1	1
neutered	18,0	M	0	0	1	0	1	1	1	0	1	1	1	1	1	1	0
intact	18,0	M	0	0	1	1	0	0	1	1	1	1	0	1	0	**1**	1
intact	18,0	M	0	0	0	0	1	0	0	0	0	1	0	1	0	**0**	1
neutered	19,0	M	0	0	1	1	1	1	0	1	1	1	0	1	0	**1**	1
intact	19,0	M	0	0	0	0	0	0	0	0	1	0	1	0	0	**1**	0
neutered	19,0	M	0	0	1	0	0	0	0	1	1	0	1	0	0	**0**	0
neutered	19,0	M	0	0	1	1	0	1	0	1	0	1	0	1	0	**1**	1
neutered	20,0	L	0	0	0	0	0	0	0	1	0	1	0	0	0	0	1
intact	20,0	L	0	0	1	0	1	0	0	1	1	0	0	1	0	**0**	0
neutered	20,0	L	0	0	1	0	0	0	0	1	0	1	1	1	0	1	0
neutered	20,0	L	0	0	1	0	0	0	0	1	0	1	1	0	0	1	0
intact	20,0	L	0	0	1	0	0	1	0	1	0	0	1	0	0	0	0
intact	20,0	L	0	0	0	0	0	0	0	1	0	1	0	1	0	0	1
intact	20,0	L	0	0	0	0	0	0	0	0	0	0	0	0	0	**0**	0
neutered	20,0	L	0	0	1	0	0	0	0	0	1	0	0	0	0	**1**	0
neutered	20,0	L	0	0	1	0	0	0	0	1	1	0	0	0	0	**1**	0
neutered	20,0	L	0	0	1	0	0	0	0	1	1	0	1	1	0	**1**	0
neutered	20,0	L	0	0	1	0	0	1	0	1	1	1	1	0	0	**1**	0
intact	20,0	L	0	0	0	0	0	0	0	0	0	1	1	1	0	**0**	0
intact	20,0	L	0	0	0	0	0	0	1	0	0	0	0	0	0	**0**	0
neutered	21,0	L	0	0	1	0	1	0	0	0	0	1	0	1	0	0	0
neutered	21,0	L	0	0	0	0	0	0	0	1	0	0	0	0	0	0	0
neutered	21,0	L	0	0	1	0	0	0	0	1	1	1	0	1	0	1	1
intact	21,0	L	0	0	1	0	1	0	0	1	1	0	0	1	1	**1**	0
neutered	21,0	L	0	0	1	1	0	0	1	1	0	1	0	0	0	**0**	0
neutered	21,0	L	0	0	1	0	1	1	0	0	1	1	1	0	0	**1**	0
intact	21,0	L	0	1	1	1	0	1	0	0	1	1	1	0	0	**1**	1
intact	22,0	L	0	0	0	0	0	0	0	0	0	1	0	0	0	**0**	0
intact	22,0	L	0	0	0	0	0	1	0	0	1	0	1	0	0	**1**	0
intact	22,0	L	0	0	0	0	0	0	0	0	0	0	0	0	0	**0**	0
neutered	22,0	L	0	0	1	0	1	0	0	1	0	1	0	1	0	1	0
neutered	22,0	L	0	0	1	0	0	0	0	1	1	0	0	0	0	1	0
neutered	22,0	L	0	0	0	0	0	0	0	0	1	1	0	0	0	0	0
intact	22,0	L	0	0	0	0	0	0	0	0	0	0	0	0	0	**0**	0
intact	22,0	L	0	0	0	0	0	0	0	1	0	0	0	0	0	**0**	0
intact	22,0	L	0	0	0	0	0	0	0	0	1	0	0	0	0	**0**	0

neutered	22,0	L	0	0	1	0	0	1	1	1	1	1	1	1	1	1	0
intact	22,4	L	0	0	1	0	0	0	1	0	1	1	0	0	0	1	0
intact	23,0	L	0	1	1	1	0	1	1	0	1	0	1	0	0	1	0
neutered	23,0	L	0	0	1	0	0	1	0	0	1	0	1	0	0	1	0
intact	23,0	L	0	0	0	0	0	0	1	0	0	0	0	0	0	0	1
intact	23,0	L	0	0	0	1	1	1	1	0	1	1	1	1	0	1	0
intact	24,0	L	0	0	1	0	0	0	0	1	1	1	0	0	0	0	0
intact	24,0	L	0	0	1	0	0	0	1	0	0	0	0	1	0	0	1
neutered	24,0	L	0	0	1	0	1	0	0	1	1	1	1	1	1	1	0
neutered	24,0	L	0	0	1	0	1	0	0	0	0	1	0	1	0	0	0
neutered	24,0	L	0	0	1	1	0	1	0	1	1	1	1	1	0	1	1
intact	24,0	L	0	0	0	0	0	0	0	1	0	0	0	0	0	0	0
intact	24,5	L	0	1	1	1	0	0	0	0	1	0	0	0	0	0	1
neutered	25,0	L	0	0	1	0	0	0	0	0	1	0	1	0	0	0	0
neutered	25,0	L	0	0	1	0	0	0	1	0	0	0	0	0	0	0	0
intact	25,0	L	0	0	1	0	0	0	0	1	0	0	0	1	1	0	1
neutered	25,0	L	0	0	0	0	0	0	1	0	1	0	0	0	0	0	0
neutered	25,0	L	0	0	0	0	0	0	1	0	0	0	0	0	0	0	1
neutered	25,0	L	0	0	1	0	0	0	0	0	0	1	0	0	0	0	1
intact	25,0	L	0	0	1	0	1	0	0	1	1	1	1	0	0	0	0
neutered	25,0	L	0	0	1	0	1	0	0	0	0	1	0	1	1	1	0
neutered	26,0	L	0	0	0	0	0	0	1	0	0	0	0	1	0	0	0
neutered	26,0	L	0	0	0	0	0	0	1	0	0	0	0	0	0	0	0
intact	26,0	L	0	0	0	0	0	0	0	1	0	0	0	1	1	1	1
intact	26,0	L	0	0	1	0	0	0	0	1	1	1	1	1	0	0	1
neutered	26,0	L	0	0	1	1	0	0	1	1	1	1	0	0	0	1	0
neutered	26,0	L	0	0	0	0	0	0	0	1	0	1	1	0	0	1	0
neutered	26,0	L	0	0	0	0	0	0	0	0	0	1	0	0	0	0	0
intact	26,0	L	0	0	0	0	0	0	0	1	0	1	0	0	0	0	1
neutered	26,6	L	0	0	1	0	0	1	0	1	0	0	1	1	0	1	0
neutered	27,0	L	1	0	1	0	0	0	0	0	1	1	0	0	0	0	0
neutered	27,0	L	0	0	1	0	1	0	0	0	0	1	0	1	1	1	0
intact	27,0	L	0	0	1	0	0	0	0	1	0	1	1	0	0	1	1
intact	27,0	L	0	0	0	0	0	0	0	0	1	0	0	1	0	0	0
neutered	27,0	L	0	0	0	0	0	1	0	1	0	0	1	1	0	1	0
neutered	27,0	L	0	0	1	0	0	0	0	0	1	1	1	1	0	1	0
intact	27,0	L	0	0	0	0	0	0	0	1	1	0	0	0	0	0	0
intact	27,0	L	0	0	1	0	1	0	0	1	0	0	0	1	0	0	1
intact	27,0	L	0	0	0	0	0	0	1	1	1	1	0	1	0	1	0
intact	27,0	L	0	0	1	0	0	1	0	0	0	1	1	0	0	0	0
intact	27,0	L	0	0	1	0	0	0	1	1	0	0	1	1	0	1	0
intact	27,0	L	0	0	1	0	0	0	0	0	0	1	0	0	0	0	1
neutered	28,0	L	0	0	0	0	0	0	0	0	1	0	0	1	0	0	0
neutered	28,0	L	0	0	1	0	1	0	0	1	1	1	0	1	1	1	0
neutered	28,0	L	0	1	1	0	0	0	0	0	0	0	0	0	0	0	0
neutered	29,0	L	0	0	1	0	0	0	1	0	0	0	0	0	0	0	1
intact	29,0	L	0	0	1	0	0	1	0	1	0	0	1	0	0	1	0

intact	29,0	L	0	0	0	0	0	0	0	0	0	1	0	0	0	**0**	0
neutered	29,0	L	0	0	0	0	0	0	0	1	0	0	0	0	0	**0**	0
neutered	29,9	L	0	1	0	0	0	0	0	1	0	0	0	0	0	**1**	1
neutered	30,0	L	0	0	0	0	0	0	0	0	1	0	0	0	0	0	0
neutered	30,0	L	0	0	1	0	0	0	0	1	0	0	1	1	0	1	0
intact	30,0	L	0	0	0	0	0	0	0	0	1	0	0	1	0	0	1
neutered	30,0	L	0	0	1	1	0	0	0	0	0	1	0	1	0	0	0
intact	30,0	L	0	0	1	0	0	0	0	0	1	1	0	0	0	**1**	1
intact	30,0	L	0	0	0	0	0	0	0	0	0	0	0	0	0	**0**	1
neutered	30,0	L	0	0	1	0	0	0	0	1	0	1	0	0	0	**0**	0
neutered	30,0	L	0	0	0	0	0	0	0	0	0	0	0	0	1	**0**	0
intact	30,0	L	0	0	0	0	0	0	0	1	0	1	0	0	0	**0**	0
intact	30,0	L	0	0	0	0	0	0	0	0	1	0	0	0	0	**0**	0
neutered	30,0	L	0	0	1	0	0	0	0	1	0	1	0	0	0	**0**	0
intact	30,0	L	0	0	0	0	0	0	0	1	0	0	0	0	0	**0**	0
neutered	30,0	L	0	0	1	0	1	0	0	1	0	0	0	1	1	**0**	1
neutered	31,0	XL	0	0	0	0	0	0	0	1	0	0	0	0	0	0	0
neutered	31,0	XL	0	0	1	1	1	1	0	1	1	0	0	1	1	1	0
intact	31,0	XL	0	0	0	0	0	1	0	0	1	0	1	0	0	**0**	0
intact	31,0	XL	0	0	0	0	0	0	0	1	0	0	0	0	0	**0**	1
intact	32,0	XL	0	0	0	0	0	1	0	0	1	0	1	0	0	0	0
intact	32,0	XL	0	0	0	0	0	0	0	1	0	0	0	0	0	0	0
neutered	32,0	XL	0	0	1	0	0	1	0	1	0	0	0	0	0	1	0
neutered	32,0	XL	0	0	1	0	0	0	1	1	0	0	0	0	0	**0**	0
intact	32,0	XL	0	0	1	0	0	1	0	0	1	1	0	0	0	**1**	0
intact	32,0	XL	0	0	0	0	0	0	0	0	1	0	0	1	1	**0**	0
neutered	32,0	XL	0	0	1	0	0	1	0	1	0	1	1	0	0	**0**	1
intact	32,0	XL	0	0	0	0	0	0	0	1	0	0	0	0	0	**0**	0
intact	33,0	XL	0	0	0	0	0	0	0	1	0	1	0	0	0	0	0
neutered	33,0	XL	0	0	0	0	0	0	0	1	0	0	1	0	1	1	0
intact	34,0	XL	0	1	0	0	0	0	0	1	0	0	0	1	0	**0**	0
intact	34,0	XL	0	0	1	1	0	0	0	0	0	1	1	0	0	1	1
intact	34,0	XL	0	0	0	0	0	0	0	1	0	0	0	0	0	**0**	0
intact	34,0	XL	0	0	0	0	0	0	0	1	0	1	0	0	0	**0**	0
neutered	34,0	XL	0	0	1	0	0	1	1	1	1	1	1	1	1	**1**	0
neutered	35,0	XL	0	0	1	0	0	0	0	1	0	1	1	0	0	0	0
intact	35,0	XL	0	0	0	0	0	0	0	0	0	1	0	0	0	0	1
intact	35,0	XL	0	0	0	0	0	0	0	0	0	0	0	0	0	**0**	0
intact	35,0	XL	0	0	0	0	0	0	0	0	0	0	0	0	0	**0**	0
intact	35,0	XL	0	0	0	0	0	0	0	1	0	0	0	0	0	**0**	1
neutered	36,0	XL	0	0	1	0	0	0	1	0	0	1	0	1	0	0	0
intact	36,0	XL	0	0	0	0	0	1	0	0	1	1	0	1	0	1	0
intact	36,0	XL	0	0	1	0	0	0	1	1	0	0	0	0	0	**0**	0
neutered	37,0	XL	0	0	0	0	0	0	0	1	0	0	0	1	0	**0**	0
neutered	39,0	XL	0	0	0	0	0	0	0	1	0	1	0	0	0	**0**	0
neutered	45,0	XL	0	0	0	0	1	1	1	1	0	0	1	1	0	1	0
neutered	45,0	XL	0	0	0	0	0	0	0	0	1	1	1	0	0	0	0

Case Studies

Neuter Status	weight (kg)	weight class	tremble	panting	Licking_ scratching	stereo- typies	Destroying objects	Barking/whining	Not house clean?	Pulling on leash	strong begging?	Aggr_Dogs	Aggr_humans	Aggr_ humans household	Aggression_Else
neutered	3,0	S	0	0	0	0	0	0	0	0	0	0	0	0	0
neutered	3,0	S	0	0	1	0	0	0	0	0	0	0	0	0	0
intact	3,1	S	0	0	0	0	0	0	0	0	0	0	0	0	0
neutered	3,9	S	1	0	0	0	0	1	1	0	0	1	1	0	0
neutered	4,0	S	1	0	1	0	0	0	0	0	0	0	0	0	0
neutered	4,0	S	1	0	0	0	0	1	0	0	0	0	0	0	0
intact	4,3	S	1	0	1	0	1	1	1	0	1	0	0	0	0
intact	5,0	S	0	0	0	0	0	0	0	0	0	0	0	0	0
neutered	5,0	S	0	1	0	0	0	1	0	0	0	1	1	0	0
intact	5,0	S	1	0	0	0	0	0	0	0	0	0	0	0	0
intact	5,0	S	0	0	0	0	1	0	0	0	0	0	0	0	0
neutered	6,0	S	1	0	0	0	0	1	0	0	0	1	0	0	0
neutered	6,0	S	1	1	1	0	1	1	0	1	0	1	0	1	0
intact	6,0	S	0	0	0	0	1	1	1	1	1	0	0	0	1
neutered	6,0	S	0	1	0	0	0	0	0	0	0	0	0	0	0
neutered	6,0	S	1	0	1	0	1	1	0	1	1	1	1	0	1
intact	6,0	S	0	0	0	0	1	1	1	0	0	0	0	0	0
intact	7,0	S	0	0	0	0	0	1	0	0	0	0	0	0	0
intact	7,0	S	1	0	0	0	0	1	0	0	0	0	0	0	0
neutered	7,0	S	1	0	1	0	0	1	0	1	1	0	0	0	0
intact	7,0	S	1	0	1	0	0	1	0	0	1	0	0	0	0
intact	7,0	S	1	0	0	0	0	1	0	1	0	0	0	0	0
neutered	7,0	S	0	1	0	0	0	0	0	1	1	0	0	0	0
intact	7,2	S	0	0	0	0	0	0	0	0	0	1	0	0	0
neutered	7,3	S	0	0	0	0	0	1	0	0	0	0	0	0	0
intact	7,5	S	1	1	1	1	0	1	0	1	1	0	1	0	0
intact	7,6	S	1	0	0	0	1	0	0	0	0	0	0	0	0
neutered	7,8	S	0	0	0	0	0	0	0	0	0	0	0	0	0
intact	8,0	S	0	0	0	0	1	0	0	0	1	0	0	0	0
neutered	8,0	S	0	0	0	1	1	0	0	0	1	0	0	0	0

neutered	8,0	S	1	1	1	0	0	1	0	1	1	1	0	0	0
neutered	8,1	S	0	0	0	0	1	1	1	1	1	1	0	0	0
intact	8,2	S	1	1	0	0	1	1	0	1	1	1	0	0	0
intact	8,5	S	0	1	0	0	0	0	0	0	0	0	0	0	0
intact	8,9	S	0	0	0	0	0	1	0	1	0	1	0	0	0
neutered	9,0	S	0	0	0	0	0	0	0	0	0	0	0	0	0
intact	9,0	S	1	0	1	0	0	1	0	0	0	1	0	0	0
intact	9,0	S	0	0	0	0	0	1	0	1	0	0	0	0	0
neutered	9,0	S	0	1	1	0	0	0	0	0	0	0	0	0	0
intact	9,5	S	0	0	0	0	0	1	0	1	0	1	0	0	0
neutered	9,5	S	1	0	1	0	0	0	0	1	1	0	0	0	0
intact	9,6	S	0	0	0	0	0	0	0	0	1	1	1	0	0
neutered	9,8	S	0	0	0	0	0	0	0	0	0	0	0	0	0
neutered	10,0	M	0	0	0	0	0	0	0	0	0	0	0	0	0
intact	10,0	M	1	0	0	0	0	1	1	1	1	0	0	0	0
intact	10,0	M	0	0	0	0	1	1	0	1	1	1	0	0	0
intact	10,0	M	0	0	0	0	0	0	0	0	0	0	0	0	0
neutered	10,0	M	0	0	1	0	0	0	0	0	0	1	0	0	0
neutered	10,0	M	1	0	1	0	1	1	0	1	1	1	1	0	0
neutered	10,0	M	0	0	0	0	0	0	0	0	0	1	0	0	0
neutered	10,0	M	1	0	0	0	0	1	0	0	1	1	1	1	0
intact	11,0	M	0	0	0	0	0	0	0	0	0	0	0	0	0
intact	11,0	M	1	1	0	0	0	0	0	1	0	0	0	0	0
neutered	11,0	M	1	0	0	0	0	0	0	1	1	1	1	0	1
intact	11,0	M	1	1	1	0	0	1	0	1	0	1	0	0	0
neutered	11,0	M	1	0	1	1	1	1	0	1	1	1	0	0	0
intact	11,0	M	1	0	1	0	0	1	0	1	1	1	1	1	0
intact	11,0	M	1	0	1	0	1	0	0	0	1	0	0	0	0
neutered	11,5	M	0	1	1	0	0	1	0	1	1	0	0	0	0
intact	12,0	M	0	0	0	0	0	0	0	0	1	0	0	0	0
neutered	12,0	M	1	0	1	1	0	1	0	0	0	1	1	0	0
neutered	12,0	M	0	0	0	0	0	1	0	0	0	1	0	0	0
intact	12,0	M	0	0	0	1	0	1	0	1	0	0	0	0	0

neutered	12,0	M	1	0	0	0	0	0	0	0	0	0	0	0	0
intact	12,0	M	0	0	1	0	1	0	0	0	1	0	0	0	0
neutered	12,0	M	1	0	1	0	0	0	0	1	1	1	1	0	0
neutered	12,0	M	0	0	0	0	0	1	0	0	0	1	0	0	0
intact	12,0	M	1	1	1	0	0	1	0	0	1	0	1	0	0
neutered	13,0	M	1	0	1	0	0	1	0	0	1	1	0	0	0
neutered	13,0	M	1	1	0	0	0	0	0	0	0	0	0	0	0
intact	13,0	M	0	1	0	0	0	1	0	0	0	1	1	0	1
intact	13,0	M	0	0	0	0	0	0	0	0	1	1	0	0	0
intact	13,0	M	0	0	0	0	0	1	0	0	1	1	0	0	0
intact	13,0	M	0	0	1	0	0	1	0	0	1	0	0	0	0
intact	13,0	M	0	0	1	0	0	0	0	0	0	0	0	0	0
intact	13,5	M	0	0	0	0	0	1	0	0	0	0	0	0	0
intact	13,5	M	0	0	0	0	0	0	0	0	0	0	0	0	0
neutered	14,0	M	1	0	0	0	0	0	0	1	0	0	0	0	0
neutered	14,0	M	0	0	0	0	0	0	0	0	1	1	1	0	0
neutered	14,0	M	1	0	1	0	0	1	0	1	1	0	0	0	0
neutered	14,0	M	0	0	0	0	0	0	0	0	1	0	0	0	0
intact	14,0	M	0	1	1	0	0	0	0	0	0	0	0	0	0
neutered	14,5	M	0	1	0	0	0	0	0	0	0	0	0	0	0
intact	14,5	M	0	0	0	0	0	1	0	0	1	0	0	0	0
intact	15,0	M	0	0	0	0	0	0	0	0	0	0	0	0	0
neutered	15,0	M	0	0	0	0	0	0	1	0	0	1	1	0	0
neutered	15,0	M	0	0	0	0	1	1	0	1	0	0	0	0	0
intact	15,1	M	1	1	1	0	0	1	0	1	1	1	0	0	0
neutered	16,0	M	1	0	1	0	0	0	0	0	1	0	0	0	0
neutered	16,0	M	0	0	0	0	0	0	0	0	0	0	0	0	0
intact	16,0	M	0	0	0	0	0	1	0	1	1	1	1	0	0
neutered	16,0	M	0	1	0	0	0	0	0	1	0	0	0	0	0
neutered	16,0	M	0	0	0	0	0	0	0	0	0	1	0	0	0
intact	17,0	M	0	0	0	0	0	0	0	0	0	0	0	0	0
intact	17,0	M	0	1	1	0	1	1	0	1	1	1	1	0	0
intact	17,0	M	1	0	1	0	0	1	0	0	0	0	0	0	0

neutered	18,0	M	0	0	0	0	0	0	0	0	0	1	1	0	0
intact	18,0	M	0	0	0	0	0	0	0	1	1	0	0	0	0
neutered	18,0	M	0	0	0	0	0	0	0	0	0	0	0	0	0
intact	18,0	M	0	0	1	1	1	1	0	1	1	1	1	0	0
neutered	18,0	M	1	0	1	0	0	0	0	1	1	1	1	1	0
intact	18,0	M	1	1	0	0	0	0	0	1	0	1	1	0	0
intact	18,0	M	1	1	1	0	1	1	0	1	1	0	0	0	0
neutered	19,0	M	0	1	1	0	1	1	0	0	1	1	0	0	0
intact	19,0	M	0	0	1	0	0	1	0	1	0	0	1	0	1
neutered	19,0	M	0	0	0	0	0	0	0	0	0	1	1	0	0
neutered	19,0	M	0	1	0	0	0	0	0	0	0	1	1	0	0
neutered	20,0	L	0	0	1	0	0	0	0	1	1	0	0	0	0
intact	20,0	L	0	0	0	0	1	1	0	1	1	0	0	0	0
neutered	20,0	L	1	1	1	0	0	1	1	1	1	1	0	0	1
neutered	20,0	L	0	1	1	0	0	0	0	0	0	0	0	0	0
intact	20,0	L	0	0	0	0	0	0	0	0	0	1	0	0	0
intact	20,0	L	0	1	0	0	0	1	0	0	0	0	0	0	0
intact	20,0	L	0	0	0	0	0	1	0	0	0	0	0	0	0
neutered	20,0	L	0	0	0	0	0	0	0	0	0	0	1	0	0
neutered	20,0	L	0	0	0	0	0	0	0	1	0	0	0	0	0
neutered	20,0	L	0	1	1	0	1	1	0	1	1	1	1	0	0
neutered	20,0	L	0	1	1	0	1	1	0	1	1	1	1	0	0
intact	20,0	L	0	0	0	0	0	0	0	1	0	1	0	0	0
intact	20,0	L	0	0	0	0	0	0	0	1	0	0	0	0	0
neutered	21,0	L	0	0	0	0	0	1	0	1	1	0	0	0	0
neutered	21,0	L	0	0	0	0	0	0	0	0	0	0	0	0	0
neutered	21,0	L	1	1	0	0	0	1	0	1	0	1	0	0	0
intact	21,0	L	0	0	1	0	0	1	0	0	0	0	0	0	0
neutered	21,0	L	0	0	0	0	0	0	0	0	0	0	0	0	0
neutered	21,0	L	1	1	0	0	0	1	0	1	0	1	1	0	0
intact	21,0	L	0	0	0	1	0	0	0	1	0	1	1	1	1
intact	22,0	L	0	0	0	0	1	1	0	1	0	1	0	0	0
intact	22,0	L	0	0	0	0	0	0	0	0	0	0	1	0	0

intact	22,0 L	0	0	1	0	0	0	0	0	0	0	0	0	0
neutered	22,0 L	1	1	0	0	0	1	0	1	1	0	0	0	0
neutered	22,0 L	1	0	0	0	0	0	0	0	0	1	0	0	0
neutered	22,0 L	0	1	0	0	0	0	0	0	0	0	0	0	0
intact	22,0 L	0	0	0	1	1	1	0	0	0	0	0	0	0
intact	22,0 L	0	1	0	0	0	1	0	0	1	1	0	0	0
intact	22,0 L	0	1	0	0	0	0	0	1	1	0	0	0	0
neutered	22,0 L	0	0	0	1	1	0	0	1	1	1	0	0	0
intact	22,4 L	1	1	0	0	0	0	0	0	0	0	0	0	0
intact	23,0 L	0	0	0	0	0	0	0	1	0	1	1	0	0
neutered	23,0 L	0	0	0	0	0	1	0	0	1	1	1	0	0
intact	23,0 L	0	1	0	0	0	0	0	1	1	0	0	0	0
intact	23,0 L	1	1	1	0	1	1	1	1	0	0	1	1	0
intact	24,0 L	0	0	0	0	0	1	0	0	1	0	0	0	0
intact	24,0 L	0	0	0	0	0	0	0	0	0	0	0	0	0
neutered	24,0 L	0	1	1	1	0	1	0	1	0	1	1	0	0
neutered	24,0 L	0	0	0	0	0	0	0	0	1	0	0	0	0
neutered	24,0 L	1	0	0	1	0	1	0	1	0	1	1	0	0
intact	24,0 L	0	0	0	0	0	0	0	0	0	0	0	0	0
intact	24,5 L	1	1	0	1	0	1	0	0	1	0	0	0	0
neutered	25,0 L	0	0	0	0	0	0	0	0	0	0	0	0	0
neutered	25,0 L	1	0	0	0	0	0	0	0	0	0	0	0	0
intact	25,0 L	0	0	1	0	0	1	0	1	1	0	0	0	0
neutered	25,0 L	0	0	0	0	0	1	0	0	0	0	0	0	0
neutered	25,0 L	0	1	1	0	0	0	0	0	1	1	0	0	0
neutered	25,0 L	0	0	1	0	1	0	0	1	1	0	0	0	0
intact	25,0 L	0	0	0	0	0	1	0	0	1	1	0	0	0
neutered	25,0 L	1	1	0	0	0	0	0	0	0	0	0	0	0
neutered	26,0 L	0	0	0	0	0	0	0	0	0	0	0	0	0
neutered	26,0 L	0	0	0	0	0	0	0	0	0	0	0	0	0
intact	26,0 L	1	0	0	0	0	1	0	1	1	1	0	0	0
intact	26,0 L	0	1	1	0	0	1	0	0	0	1	0	0	0
neutered	26,0 L	1	1	0	0	0	1	0	0	0	0	1	0	1

neutered	26,0 L	0	0	0	0	0	1	0	1	0	**1**	**0**	**0**	**1**
neutered	26,0 L	0	0	0	1	0	0	0	1	0	**0**	**0**	**0**	**0**
intact	26,0 L	0	1	0	0	1	1	1	1	1	**0**	**0**	**0**	**1**
neutered	26,6 L	0	1	1	1	0	0	0	0	1	**1**	**0**	**0**	**0**
neutered	27,0 L	0	1	1	0	1	1	0	0	0	0	0	0	0
neutered	27,0 L	0	1	1	0	0	0	0	0	1	0	0	0	0
intact	27,0 L	0	0	0	0	0	1	0	1	0	1	0	0	0
intact	27,0 L	0	0	0	0	0	0	0	1	1	0	0	0	0
neutered	27,0 L	0	1	0	0	0	0	0	0	0	1	1	0	0
neutered	27,0 L	0	0	0	0	0	1	0	1	0	**0**	**0**	**0**	**1**
intact	27,0 L	0	0	0	0	0	1	0	0	1	**0**	**0**	**0**	**0**
intact	27,0 L	0	1	0	0	0	0	0	1	0	**0**	**0**	**0**	**0**
intact	27,0 L	0	0	1	0	1	0	0	0	0	**0**	**0**	**0**	**0**
intact	27,0 L	0	1	1	0	0	1	1	1	0	**0**	**0**	**0**	**0**
intact	27,0 L	0	0	0	0	0	0	0	1	0	**0**	**0**	**0**	**0**
intact	27,0 L	0	0	0	0	0	0	0	0	0	**1**	**1**	**0**	**0**
neutered	28,0 L	1	0	0	0	0	0	0	0	0	0	1	0	0
neutered	28,0 L	1	0	0	0	0	1	0	1	1	**0**	**0**	**0**	**0**
neutered	28,0 L	0	1	0	0	0	0	0	0	0	**0**	**0**	**0**	**0**
neutered	29,0 L	0	0	0	0	0	0	0	0	0	0	0	0	0
intact	29,0 L	0	0	0	0	0	1	0	0	0	1	1	0	0
intact	29,0 L	0	0	0	0	0	0	0	0	0	**0**	**0**	**0**	**0**
neutered	29,0 L	0	1	0	0	0	0	0	0	0	**0**	**0**	**0**	**0**
neutered	29,9 L	0	0	0	0	0	1	0	1	0	**0**	**0**	**0**	**0**
neutered	30,0 L	0	0	0	0	0	0	0	0	0	0	0	0	0
neutered	30,0 L	0	0	0	0	0	1	0	1	1	1	0	0	0
intact	30,0 L	0	0	0	0	0	1	0	0	0	0	0	0	0
neutered	30,0 L	0	1	1	0	0	0	0	0	0	0	0	0	0
intact	30,0 L	1	1	0	0	0	0	0	1	0	**0**	**0**	**0**	**0**
intact	30,0 L	0	0	1	0	0	0	0	0	1	**0**	**0**	**0**	**0**
neutered	30,0 L	0	0	0	0	0	0	0	0	0	**1**	**0**	**0**	**0**
neutered	30,0 L	0	0	0	0	0	0	0	0	0	**1**	**0**	**0**	**0**
intact	30,0 L	0	0	0	0	0	0	0	1	0	**1**	**0**	**0**	**0**

intact	30,0	L	0	0	0	0	1	1	0	1	0	**0**	**0**	**0**	**0**
neutered	30,0	L	1	1	0	0	0	1	0	0	0	**0**	**0**	**0**	**0**
intact	30,0	L	0	0	0	0	0	0	0	0	0	**0**	**0**	**0**	**0**
neutered	30,0	L	0	0	1	0	1	1	0	1	1	**0**	**0**	**0**	**0**
neutered	31,0	XL	0	0	0	0	0	0	0	0	0	0	0	0	0
neutered	31,0	XL	1	0	1	1	1	1	0	0	1	1	1	1	0
intact	31,0	XL	0	1	0	0	0	0	1	0	0	**1**	**1**	**1**	**0**
intact	31,0	XL	1	1	0	0	0	0	0	1	0	**0**	**0**	**0**	**0**
intact	32,0	XL	0	0	0	0	0	0	0	0	0	0	1	0	0
intact	32,0	XL	0	0	0	0	0	0	0	0	0	0	0	0	0
neutered	32,0	XL	1	0	0	0	0	1	0	1	0	1	0	0	0
neutered	32,0	XL	0	1	0	0	1	0	0	0	0	**0**	**0**	**0**	**0**
intact	32,0	XL	1	0	1	0	0	0	0	0	0	**1**	**0**	**0**	**0**
intact	32,0	XL	0	0	0	0	0	0	0	0	1	**1**	**0**	**0**	**0**
neutered	32,0	XL	0	1	0	0	0	1	0	1	1	**1**	**0**	**0**	**1**
intact	32,0	XL	0	0	0	0	0	0	0	0	0	**0**	**0**	**0**	**0**
intact	33,0	XL	0	0	0	0	0	1	0	0	0	0	0	0	0
neutered	33,0	XL	1	1	0	0	0	1	0	0	0	1	0	0	0
intact	34,0	XL	0	1	1	0	0	1	0	0	1	**0**	**0**	**0**	**0**
intact	34,0	XL	0	1	0	0	0	0	0	1	0	1	0	0	0
intact	34,0	XL	0	0	0	0	1	0	0	1	0	**0**	**0**	**0**	**0**
intact	34,0	XL	0	0	0	0	0	1	0	1	1	**1**	**0**	**0**	**0**
neutered	34,0	XL	0	0	1	0	0	0	0	1	1	**1**	**1**	**0**	**1**
neutered	35,0	XL	0	0	0	0	0	0	0	0	0	1	0	0	0
intact	35,0	XL	0	0	1	0	0	1	0	1	1	0	0	0	0
intact	35,0	XL	0	0	0	0	0	0	0	0	0	**0**	**0**	**0**	**0**
intact	35,0	XL	0	1	0	0	0	0	0	0	0	**0**	**0**	**0**	**0**
intact	35,0	XL	1	0	0	0	0	0	0	0	0	**0**	**0**	**0**	**0**
neutered	36,0	XL	0	1	0	0	0	0	0	0	1	0	0	0	0
intact	36,0	XL	1	0	0	0	1	1	0	1	1	1	0	0	0
intact	36,0	XL	1	0	0	0	0	0	0	0	1	**0**	**0**	**0**	**0**
neutered	37,0	XL	0	1	0	1	0	1	0	1	0	**0**	**0**	**0**	**0**
neutered	39,0	XL	0	0	0	0	0	0	0	0	0	**0**	**0**	**0**	**0**

neutered	45,0	XL	1	1	0	0	1	0	0	0	0	1	0	0	0
neutered	45,0	XL	0	0	0	0	0	0	0	0	0	0	0	0	0

BUDAPEST QUESTIONNAIRE							
ID	Neuter Status	Weight (kg)	Weight class	Emotional stability	Trainability	Sociability	Extraversion
69921932	neutered	3,0	S	8	7	7	6
81264969	neutered	3,0	S	8	8	6	5
69160432	intact	3,1	S	5	9	7	0
75549299	neutered	3,9	S	2	10	6	4
68644438	neutered	4,0	S	3	0	6	0
76972132	neutered	4,0	S	5	7	6	3
77730733	intact	4,3	S	4	9	8	4
68591363	intact	5,0	S	6	9	6	5
69129235	neutered	5,0	S	3	5	2	3
69405898	intact	5,0	S	4	9	8	1
70491950	intact	5,0	S	5	7	7	4
113	neutered	6,0	S	3	10	5	5
69137534	neutered	6,0	S	0	7	1	5
75196510	intact	6,0	S	0	8	7	3
77074251	neutered	6,0	S	3	10	5	6
77721424	neutered	6,0	S	0	10	1	6
77728315	intact	6,0	S	1	9	7	4
75179101	intact	7,0	S	8	10	7	5
75327091	intact	7,0	S	5	9	8	4
77732421	neutered	7,0	S	6	8	7	5
77738238	intact	7,0	S	0	9	5	5
77786262	intact	7,0	S	5	7	5	5
77790493	neutered	7,0	S	3	7	3	5
59	intact	7,2	S	8	4	4	6
70045607	neutered	7,3	S	5	6	3	3
74233005	intact	7,5	S	2	1	4	2
70472810	intact	7,6	S	1	10	8	5
77789040	neutered	7,8	S	6	10	7	5
77721266	intact	8,0	S	1	10	7	5
77728419	neutered	8,0	S	7	9	8	6
77757873	neutered	8,0	S	0	7	1	2
77788346	neutered	8,1	S	1	8	2	6
77733284	intact	8,2	S	5	10	5	6
77811121	intact	8,5	S	2	10	7	6
77754983	intact	8,9	S	7	10	3	4
161	neutered	9,0	S	7	7	6	4
185	intact	9,0	S	4	7	4	5
73803050	intact	9,0	S	4	9	6	2
74007931	neutered	9,0	S	3	3	4	0
71487101	intact	9,5	S	8	10	4	6
77782996	neutered	9,5	S	1	9	5	4
73807319	intact	9,6	S	2	10	1	1
77720739	neutered	9,8	S	5	8	8	6
183	neutered	10,0	M	7	2	6	5
70063135	intact	10,0	M	5	6	8	6
72334147	intact	10,0	M	5	9	5	6
72876685	intact	10,0	M	7	10	6	6
73812562	neutered	10,0	M	6	7	3	6

77727875	neutered	10,0	M	1	7	2	3
77728754	neutered	10,0	M	0	7	4	5
77738340	neutered	10,0	M	3	4	2	6
157	intact	11,0	M	3	9	7	4
70083534	intact	11,0	M	3	10	7	5
75552195	neutered	11,0	M	1	8	2	3
77729311	intact	11,0	M	0	4	0	5
77729954	neutered	11,0	M	5	1	5	3
77731870	intact	11,0	M	0	10	4	6
77744028	intact	11,0	M	4	10	8	6
72	neutered	11,5	M	2	8	6	5
69678259	intact	12,0	M	4	9	7	5
73801672	neutered	12,0	M	0	8	0	6
75544064	neutered	12,0	M	6	5	2	1
75554507	intact	12,0	M	6	9	8	6
77717078	neutered	12,0	M	8	7	5	1
77736702	intact	12,0	M	6	10	8	6
77754104	neutered	12,0	M	3	3	0	4
77773159	neutered	12,0	M	4	9	2	2
77776954	intact	12,0	M	4	9	8	1
165	neutered	13,0	M	1	3	0	0
69290383	neutered	13,0	M	6	10	7	1
70810212	intact	13,0	M	2	10	4	5
72621985	intact	13,0	M	5	10	5	6
77719841	intact	13,0	M	5	10	4	6
77726492	intact	13,0	M	4	7	8	4
77730774	intact	13,0	M	0	9	7	6
70833173	intact	13,5	M	4	9	8	5
77729127	intact	13,5	M	7	10	8	6
144	neutered	14,0	M	1	8	8	1
152	neutered	14,0	M	0	8	3	4
77712502	neutered	14,0	M	1	10	3	4
77719482	neutered	14,0	M	2	7	7	2
77803914	intact	14,0	M	6	9	7	5
75544188	neutered	14,5	M	1	8	7	3
77753301	intact	14,5	M	0	9	3	4
69396188	intact	15,0	M	6	5	7	5
70043670	neutered	15,0	M	2	6	0	6
77797740	neutered	15,0	M	5	6	8	1
77722009	intact	15,1	M	0	9	5	5
34	neutered	16,0	M	1	9	7	6
63	neutered	16,0	M	1	5	5	0
184	intact	16,0	M	0	8	5	5
69381993	neutered	16,0	M	7	7	3	2
75544003	neutered	16,0	M	4	7	2	3
69189303	intact	17,0	M	2	10	8	6
75577094	intact	17,0	M	0	10	4	4
77736472	intact	17,0	M	4	9	5	5
25	neutered	18,0	M	0	6	4	3
111	intact	18,0	M	2	7	6	5

160	neutered	18,0	M	6	2	5	1
164	intact	18,0	M	1	10	8	5
187	neutered	18,0	M	0	8	2	5
73802533	intact	18,0	M	0	4	4	1
81686115	intact	18,0	M	3	9	8	5
69162090	neutered	19,0	M	0	9	7	4
69167886	intact	19,0	M	1	5	6	3
75551985	neutered	19,0	M	3	9	3	4
80193622	neutered	19,0	M	1	7	6	6
28	neutered	20,0	M	4	8	8	3
37	intact	20,0	M	2	9	8	6
95	neutered	20,0	M	0	3	3	4
105	neutered	20,0	M	0	5	1	0
156	intact	20,0	M	4	7	7	4
180	intact	20,0	M	4	5	7	4
70081414	intact	20,0	M	6	7	8	3
70834551	neutered	20,0	M	4	9	6	2
70849510	neutered	20,0	M	2	6	8	6
75544849	neutered	20,0	M	2	10	6	4
77719585	neutered	20,0	M	3	9	8	6
77731481	intact	20,0	M	4	10	5	6
77738931	intact	20,0	M	4	10	6	2
75	neutered	21,0	L	3	8	8	6
138	neutered	21,0	L	3	8	2	5
182	neutered	21,0	L	4	10	6	2
69135119	intact	21,0	L	1	6	2	6
69138036	neutered	21,0	L	2	9	7	4
75554010	neutered	21,0	L	0	10	8	6
80560946	intact	21,0	L	0	8	4	1
23	intact	22,0	L	5	8	8	6
29	intact	22,0	L	4	10	6	3
60	intact	22,0	L	8	9	8	5
121	neutered	22,0	L	0	9	7	6
146	neutered	22,0	L	2	5	4	4
181	neutered	22,0	L	2	9	6	2
70089102	intact	22,0	L	4	10	6	2
72625748	intact	22,0	L	1	10	5	6
77774758	intact	22,0	L	6	8	4	3
81068352	neutered	22,0	L	3	10	4	3
81447614	intact	22,4	L	1	6	6	2
19	intact	23,0	L	0	10	7	5
70	neutered	23,0	L	0	8	6	6
167	intact	23,0	L	4	10	7	6
68582659	intact	23,0	L	1	2	4	6
62	intact	24,0	L	3	10	8	3
72622857	intact	24,0	L	0	5	8	5
73811587	neutered	24,0	L	0	7	3	5
73815316	neutered	24,0	L	3	8	7	5
73923640	neutered	24,0	L	0	10	2	3
79950034	intact	24,0	L	7	5	6	4

75544611	intact	24,5	L	4	7	6	2
33	neutered	25,0	L	2	4	6	5
139	neutered	25,0	L	4	4	7	5
69169231	intact	25,0	L	1	7	8	5
69429570	neutered	25,0	L	3	9	8	6
74888716	neutered	25,0	L	3	6	4	0
75549246	neutered	25,0	L	2	3	7	4
76355934	intact	25,0	L	2	10	7	6
77785404	neutered	25,0	L	1	7	3	4
141	neutered	26,0	L	1	9	4	6
169	neutered	26,0	L	8	9	6	1
69674489	intact	26,0	L	5	10	7	6
72745100	intact	26,0	L	5	8	6	5
73781232	neutered	26,0	L	1	7	2	3
73813086	neutered	26,0	L	2	10	3	6
75583837	neutered	26,0	L	1	3	5	6
79904066	intact	26,0	L	6	7	8	6
75698830	neutered	26,6	L	0	5	4	4
22	neutered	27,0	L	6	4	6	0
112	neutered	27,0	L	1	8	6	6
142	intact	27,0	L	1	10	6	6
	intact	27,0	L	2	10	7	4
168	neutered	27,0	L	0	6	0	6
69132271	neutered	27,0	L	1	10	2	4
69136475	intact	27,0	L	6	8	6	5
73527505	intact	27,0	L	6	9	7	4
77712970	intact	27,0	L	0	10	7	5
77736265	intact	27,0	L	2	5	7	3
77767366	intact	27,0	L	0	8	6	6
81608288	intact	27,0	L	1	1	5	3
79	neutered	28,0	L	4	6	6	4
77730137	neutered	28,0	L	1	10	7	5
77782759	neutered	28,0	L	4	3	4	2
13	neutered	29,0	L	7	0	5	1
151	intact	29,0	L	2	7	2	6
70438063	intact	29,0	L	7	10	7	3
77783045	neutered	29,0	L	8	9	7	6
74806405	neutered	29,9	L	1	9	8	6
117	neutered	30,0	L	5	9	8	3
147	neutered	30,0	L	5	5	6	5
155	intact	30,0	L	1	7	7	0
166	neutered	30,0	L	2	3	5	4
69396256	intact	30,0	L	2	8	5	6
70321294	intact	30,0	L	6	10	8	6
72786930	neutered	30,0	L	8	6	6	2
77417320	neutered	30,0	L	4	10	2	6
77784648	intact	30,0	L	7	8	6	4
77787310	intact	30,0	L	3	10	7	5
77813488	neutered	30,0	L	3	8	6	2
77827886	intact	30,0	L	6	8	7	6

79295239	neutered	30,0	L	5	6	8	5
129	neutered	31,0	XL	6	3	7	2
131	neutered	31,0	XL	1	7	4	6
77730341	intact	31,0	XL	6	1	1	4
81687965	intact	31,0	XL	6	5	6	6
84	intact	32,0	XL	7	7	6	3
135	intact	32,0	XL	6	10	7	5
186	neutered	32,0	XL	6	8	8	6
70287554	neutered	32,0	XL	2	10	8	6
77754806	intact	32,0	XL	1	6	0	3
77779616	intact	32,0	XL	6	7	6	6
77789720	neutered	32,0	XL	1	8	7	6
78654008	intact	32,0	XL	5	8	7	6
132	intact	33,0	XL	4	8	8	2
153	neutered	33,0	XL	1	8	5	6
30	intact	34,0	XL	2	8	7	2
179	intact	34,0	XL	1	3	4	2
70078916	intact	34,0	XL	5	8	7	6
72851461	intact	34,0	XL	8	9	6	6
81699336	neutered	34,0	XL	1	9	3	6
114	neutered	35,0	XL	1	9	3	2
176	intact	35,0	XL	3	3	6	6
68716714	intact	35,0	XL	7	10	7	3
77331166	intact	35,0	XL	6	2	8	6
77740864	intact	35,0	XL	3	3	3	2
110	neutered	36,0	XL	2	8	5	0
163	intact	36,0	XL	3	8	6	6
77737788	intact	36,0	XL	2	8	3	2
77714327	neutered	37,0	XL	5	9	4	4
73809437	neutered	39,0	XL	6	5	6	3
109	neutered	45,0	XL	0	4	2	6
123	neutered	45,0	XL	4	1	3	1
